# Integrative taxonomy of root-knot nematodes reveals multiple independent origins of mitotic parthenogenesis

**DOI:** 10.1371/journal.pone.0172190

**Published:** 2017-03-03

**Authors:** Toon Janssen, Gerrit Karssen, Olivera Topalović, Danny Coyne, Wim Bert

**Affiliations:** 1 Nematology Research Unit, Department of Biology, Ghent University, K.L. Ledeganckstraat, Ghent, Belgium; 2 Center for Medical Genetics, Reproduction and Genetics, Reproduction Genetics and Regenerative Medicine, Vrije Universiteit Brussel, UZ Brussel, Laarbeeklaan, Brussels, Belgium; 3 National Plant Protection Organization, Wageningen Nematode Collection, HC Wageningen, The Netherlands; 4 International Institute of Tropical Agriculture (IITA), c/o *icipe*, Kasarani, Nairobi, Kenya; INRA, FRANCE

## Abstract

During sampling of several *Coffea arabica* plantations in Tanzania severe root galling, caused by a root-knot nematode was observed. From pure cultures, morphology and morphometrics of juveniles and females matched perfectly with *Meloidogyne africana*, whereas morphology of the males matched identically with those of *Meloidogyne decalineata*. Based on their Cox1 sequence, however, the recovered juveniles, females and males were confirmed to belong to the same species, creating a taxonomic conundrum. Adding further to this puzzle, re-examination of *M*. *oteifae* type material showed insufficient morphological evidence to maintain its status as a separate species. Consequently, *M*. *decalineata* and *M*. *oteifae* are synonymized with *M*. *africana*, which is herewith redescribed based on results of light and scanning electron microscopy, ribosomal and mitochondrial DNA sequences, isozyme electrophoresis, along with bionomic and cytogenetic features. Multi-gene phylogenetic analysis placed *M*. *africana* outside of the three major clades, together with *M*. *coffeicola*, *M*. *ichinohei* and *M*. *camelliae*. This phylogenetic position was confirmed by several morphological features, including cellular structure of the spermatheca, egg mass position, perineal pattern and head shape. Moreover, *M*. *africana* was found to be a polyphagous species, demonstrating that “early-branching” *Meloidogyne* spp. are not as oligophagous as had previously been assumed. Cytogenetic information indicates *M*. *africana* (2n = 21) and *M*. *ardenensis* (2n = 51–54) to be a triploid mitotic parthenogenetic species, revealing at least four independent origins of mitotic parthenogenesis within the genus *Meloidogyne*. Furthermore, *M*. *mali* (n = 12) was found to reproduce by amphimixis, indicating that amphimictic species with a limited number of chromosomes are widespread in the genus, potentially reflecting the ancestral state of the genus. The wide variation in chromosome numbers and associated changes in reproduction modes indicate that cytogenetic evolution played a crucial role in the speciation of root-knot nematodes and plant-parasitic nematodes in general.

## Introduction

Coffee (*Coffea arabica* L.) is one of the most important cash crops worldwide and the second most important traded commodity after oil, with an estimated total export value of US$ 19.1 billion in 2012/2013 [1]. An estimated 100 million people worldwide are dependent on growing coffee, most of them from developing tropical countries [2]. In Africa, coffee generates substantial income for rural communities and is a primary source of income for an estimated 10 million households in 25 countries [3]. However, coffee production in Africa is declining, by approximately 17% since the 1970’s [3], while in other regions coffee production has essentially doubled over the last 50 years. Meanwhile, global coffee consumption continues to rise at an accelerating rate [1]. There are various reasons for the coffee production decline in Africa, among them losses due to pests and diseases, and the costs involved in dealing with them. Pesticides, for example, account for over 30% of coffee production costs [1, 3]. Of the various ailments that plague coffee production, plant-parasitic nematodes, in particular root-knot nematodes (*Meloidogyne* spp.), are an especially significant, yet relatively overlooked threat. In South and Central America, where most of the available information on nematode pests of coffee has been attained, nematodes are recognized as highly damaging pests, responsible for the complete destruction of coffee plantations, to the point of forcing a shift to other crops, such as sugar cane [4]. Often, coffee can only be cultivated when grafted onto nematode-resistant root-stocks.

In South and Central America the root-knot nematodes that cause damage to coffee roots are *M*. *exigua* Göldi, 1887, *M*. *incognita* (Kofoid & White, 1919) Chitwood, 1949, *M*. *coffeicola* Lordello & Zamith, 1960, *M*. *paranaensis* Carneiro, Carneiro, Abrantes, Santos & Almeida, 1996, *M*. *hapla* Chitwood, 1949, *M*. *arenaria* Chitwood, 1949, *M*. *inornata* Lordello, 1956, *M*. *arabicida* Lopez & Salazar, 1989, *M*. *konaensis* Eisenback, Bernard and Schmitt, 1994, *M*. *enterolobii* Rammah & Hirschmann 1988, *M*. *izalcoensis* Carneiro, Almeida, Gomes and Hernandez, 2005 and *M*. *lopezi* Humphreys-Pereira, Flores-Chaves, Gomez, Salazar, Gomez-Alpizar & Elling, 2014 [5, 6]. Most of these species can be identified using species-specific primers that have been developed to amplify sequence-characterized amplified regions (SCAR), having themselves been converted from diagnostic randomly amplified polymorphic DNA fragments (RAPDs) [7, 8].

Despite the importance of root-knot nematodes on coffee, there is virtually no information on nematodes of coffee in Africa [5]. *Meloidogyne* spp. reported on coffee in Africa include the widely distributed *M*. *incognita*, *M*. *javanica*, [4] and recently *M*. *izalcoensis* and *M*. *hapla* [9]. There are also five species which have been almost exclusively reported from Africa, *M*. *africana* Whitehead, 1959, *M*. *kikuyensis* De Grisse, 1960, *M*. *oteifae* Elmiligy 1968, *M*. *megadora* Whitehead, 1968 and *M*. *decalineata* Whitehead, 1968. *Meloidogyne africana* has been reported on *C*. *arabica* in Kenya [4, 10] and Tanzania [11], on *C*. *canephora* L. in Democratic Republic of Congo [12], on pepper (*Capsicum annuum* L.) in Sudan [13] and once outside of Africa on maize (*Zea mays* L.) in India [14]. *Meloidogyne kikuyensis* was originally described from *Pennisetum clandestinum* Hochst. in Kenya [15] but was also reported from coffee by Whitehead [16]. *Meloidogyne oteifae* was described from *Pueraria javanica* Benth. and *C*. *canephora* in Congo, but has not been reported since [17]. *Meloidogyne megadora* was originally described from *C*. *arabica* and *C*. *canephora* in the Republic of Angola [18] and later reported from Uganda and São Tomé and Príncipe [4, 19]. *Meloidogyne decalineata* was also reported in coffee nurseries on São Tomé Island [20].

Since the monumental taxonomical work of Whitehead [10, 16, 18], progress on the taxonomy of African root-knot nematodes has been limited. Problematically, most descriptions are only based on a limited number of morphological features [5], causing problems in species diagnostics, since morphological identification of root-knot nematodes and indeed of nematodes in general is known to be greatly hampered by phenotypic plasticity [21, 22]. As a result of this, and limited local expertise, root-knot nematode infections on coffee in Africa are rarely identified up to species level [23].

In 2013, severe root-galling was observed in several coffee plantations in the Lushoto and Mbelei districts of Tanzania. Intriguingly, initial phylogenetic analyses revealed the root-knot nematode to be outside the three classically recognised clades within the genus [24], such species are not frequently encountered in field surveys, and as a consequence, little morphological and isozymic information is available, while cytogenetic information is missing completely [25]. Interestingly, *M*. *coffeicola*, also a coffee root-knot nematode species from Brazil, is also considered to be to be outside the three well-known clades [26], while *M*. *ichinohei* Araki, 1992, a root-knot nematode from Japan parasitizing *Iris laevigata* Fisch. & Mey., 1839 [27], *M*. *camelliae* Golden, 1979 and an undescribed *Meloidogyne* species from *Sansevieria* sp. are reported occupying a paraphyletic phylogenetic position in the genus, based on ribosomal rDNA [27, 28]. However, studying oogenesis of these uncommon species would most likely allow insight into the complex cytogenetic history of the genus *Meloidogyne* [25].

Formerly, amphimictic root-knot nematodes were hypothesized to be the ancestral state within the genus *Meloidogyne*, meiotic parthenogenetic species derived from them and mitotic parthenogenetic species evolved from meiotic parthenogenetic species [29–31]. This hypothesis was primarily based on the low chromosome number of obligatory amphimictic species: *M*. *spartinae* (Rau & Fassuliotis, 1965) Whitehead, 1968 [32] and *M*. *kikuyensis* [31]. Both species have n = 7 chromosomes, while *M*. *carolinensis* Eisenback, 1982, *M*. *megatyla* Baldwin & Sasser, 1979 and *M*. *microtyla* Mulvey, Townshend & Potter, 1975 have n = 18 chromosomes [30]. However, molecular phylogenies demonstrated that *M*. *microtyla* [24] and *M*. *spartinae* [27, 33] did not occur at their assumed early diverging position, while the assumed meiotic parthenogenetic species *M*. *artiellia* Franklin, 1961 (cytology of this species was never formally studied) does take an early diverging position [25, 34, 35]. Consequently, this provided support for the alternative hypothesis of Triantaphyllou [30], in which meiotic parthenogenetic species reflect the ancestral state in comparison to amphimictic and mitotic parthenogenetic species [25]. Moreover, mitotic parthenogenetic species are reported to have several origins, one in clade I in which all species are mitotic parthenogens, except *M*. *floridensis* Handoo, Nyczepir, Esmenjaud, van der Beek, Castagnone-Sereno, Carta, Skantar & Higgins, 2004, and one in clade II in which *M*. *hapla* race B and *M*. *partityla* are described to be mitotic parthenogens [36, 37], while in in clade III *M*. *oryzae* Maas, Sanders & Dede, 1978 is the only apomictic species among facultative meiotic parthenogens [24, 25, 27].

Recovery and culturing of *M*. *africana* facilitated the current study on the evolution of reproduction and oogenesis within the genus. However, to identify this species a taxonomic conundrum needed to be resolved, based on the limited available morphological and molecular information for African coffee root-knot nematodes. Therefore, the first objective of the current study was to perform an integrative taxonomic description based on LM, SEM, four loci (18S, ITS, 28S, Cox1) and isozyme phenotyping, which remains essential for accurate diagnosis of root-knot nematodes [22]. The second objective was to gain insight into the bionomics and mode of reproduction by studying host symptoms and cytology of the parasite. The final objective was to place morphological, isozyme, bionomic and cytological findings in an evolutionary perspective using a multi-gene phylogeny of the genus *Meloidogyne* to reveal insight into the evolution of reproduction and oogenesis within the genus.

## Materials and methods

### Collection of populations, culturing and host-range test

In September 2013, root samples of *C*. *arabica* showing a characteristic root-knot galling were collected from nine fields from two villages in the West Usambaras Lushoto Mountain Reserve (Tanzania): Mbelei (-4.83166,38.39883; -4.82946,38.39694; -4.82824,38.395778; -4.83183,38.39866; -4.83025,38.399120; -04.79879,038.30156) and Lushoto (-4.79879,38.30156; -4.80096,38.29975; -04.82946,038.39694). No specific permit was required for this sampling and this study did not involve endangered species. Nematodes were extracted from soil using a modified Baermann funnel [38] and subsequently fixed in DESS solution [39]. From each sample, infected roots were directly fixed in DESS solution. Second-stage juveniles were hand-picked from dissected galls of fresh roots using a stereomicroscope and inoculated onto *C*. *arabica* seedlings in individual pots containing sterile potting media and maintained in a greenhouse of the National Plant Protection Organization (Wageningen, the Netherlands) at 23°C. Nematodes from this *C*. *arabica* culture were extracted using a variety of techniques including the modified Baermann funnel, Oostenbrink elutriator, centrifugal sugar flotation, mistifier and gall dissection [40]. A host-range test was performed by inoculating 1500 juveniles on *Sanseveiria* sp. and *Solanum lycopersicum* L. (cv. Moneymaker).

### Morphological characterization

Nematodes were studied in temporary preparations sealed with nail-polish using an Olympus BX50 DIC microscope (Olympus Optical), DESS fixed specimens were first transferred to water for 1 hour before mounting in temporary slides. Morphological vouchers were created using a combination of movies and photomicrographs with an Olympus C5060Wz camera, which are available online at http://www.nematodes.myspecies.info and on the Dryad Digital Repository (http://datadryad.org/). Vouchered nematodes were subsequently picked from temporary mounts and processed for DNA extraction. Nematodes from *C*. *arabica* cultures were fixed in TAF (Triethanolamine 2%, Formalin 7%, distilled water 91%) at 70°C and processed to anhydrous glycerine, following the method of Seinhorst [41] modified by Sohlenius and Sandor [42]. These TAF fixed specimen were used for a profound morphometric analysis. Comparative morphological analysis of each live stage as presented in section 1 are based on a combination of field caught populations and the population in culture. The cellular architecture of the gonads of egg laying females was examined after dissection in temporary mounts according to the method of Bert et al. [43]; movies of spermatheca morphology are available online on Dryad Digital Repository (http://dx.doi.org/10.5061/dryad.9f63r). For scanning electron microscopy (SEM) nematodes were fixed in 600 μl fresh 4% Paraformaldehyde fixative buffered with Phosphate Buffer Saline (PBS) and 1% glycerol and heated for 3 seconds in a 750W microwave. Subsequently specimens were dehydrated in a seven-step graded series of ethanol solutions and critical-point dried using liquid CO_2_, mounted on stubs with carbon discs, coated with gold 25 nm. Specimens were studied and photographed with a JSM-840 EM (JEOL) electron microscope at 12 kV.

### Isozyme analysis

Esterase and malate dehydrogenase isozymes were analysed according to Karssen et al. [44]. Ten young females were isolated from root cultures in isotonic (0.9%) NaCl solution. Individual females, after desalting in reagent-grade water on ice for 5 minutes, were loaded to sample wells containing 0.6 μl extraction buffer (20% sucrose, 2% triton X-100, 0.01% Bromophenol Blue), and subsequently macerated using a glass rod. This mixture was homogenized, and protein extractions were loaded onto a (8–25) polyacrylamide gradient gel and electrophoretically fractioned using a PhastSystem (Pharmacia Ltd., Uppsala, Sweden). In addition to the ten test samples, two *M*. *javanica* protein extractions were added to the centre of each gel to serve as a reference. After electrophoresis, gels were stained to examine for malate dehydrogenase (Mdh) and esterase (Est) activity for 5 and 45 minutes, respectively, rinsed with distilled water, and fixed using a 10% glycerol, 10% acetic acid, distilled water solution.

### DNA extraction, PCR amplification and sequencing

Genomic DNA was extracted from both live specimen and specimen fixed in DESS solution. Genomic DNA of individual crushed females was extracted using worm lysis buffer and proteinase K [45] while genomic DNA of juveniles and males was extracted using the quick alkaline lysis protocol described by Janssen et al. [46]. Briefly, individual nematodes were transferred to a mixture of 10 μl 0.05N NaOH and 1 μl of 4.5% Tween. The mixture was heated to 95°C for 15 min, and after cooling to room temperature 40 μl of double-distilled water was added. PCR amplification was performed using toptaq DNA polymerase (QIAGEN, Germany), in a volume of 25 μl using a Bio-Rad T100^TM^ thermocycler. PCR mixtures were prepared according to the manufacturer’s protocol with 0.4 μM of each primer and 2 μl of single nematode DNA extraction. The 28S rDNA fragment D2A (ACA AGT ACC GTG AGG GAA AGT TG) and D3B (TCG GAA GGA ACC AGC TAC TA) primers were used according to De Ley et al. [47]. The 18S rDNA gene was amplified using G18S4 (GCT TGT CTC AAA GAT TAA GCC) and 18P (TGA TCC WKC YGC AGG TTC AC) with internal sequencing primers 4F (CAA GGA CGA WAG TTW GAG G) and 4R (GTA TCT GAT CGC CKT CGA WC) according to Bert et al. [45]. The internal transcribed rDNA spacer (ITS) was amplified using VRAIN2F (CTT TGT ACA CAC CGC CCG TCG CT) and VRAIN2R (TTT CAC TCG CCG TTA CTA AGG GAA TC) subsequently cloned using pGEM ® -T easy vector systems (Promega) and sequenced using universal M13F and M13R primers. The Cytochrome c oxidase subunite 1 (Cox1) gene fragment was amplified using JB3 (TTT TTT GGG CAT CCT GAG GTT TAT) and JB4.5 (TAA AGA AAG AAC ATA ATG AAA ATG) [48, 49]. Sanger sequencing of purified PCR fragments was carried out in forward and reverse direction by Macrogen (Europe). Contigs were assembled using GENEIOUS R6.1.8 (Biomatters; http://www.Geneious.com). All contigs were subjected to BLAST searches to check for possible contaminations on http://www.ncbi.nlm.nih.gov.

### Sequence analysis

Multiple sequence alignments of single ribosomal genes (18S, 28S and ITS) were made using the Q-INS-i algorithm in MAFFT 7.271 (http://mafft.cbrc.jp/alignment/server/index.html), which accounts for the secondary structure of rRNA [50]. Cox1 sequences were translated using the TranslatorX webserver (http://translatorx.co.uk/) [51], using the invertebrate genetic code, and the nucleotides aligned according to an amino acid alignment constructed using MAFFT. Multiple sequence alignments are available on Dryad Digital Repository (http://dx.doi.org/10.5061/dryad.9f63r). Post alignment trimming was conducted using the parametric profiling method of ALISCORE2.2 [52]. Gaps were treated as a 5^th^ character and the default sliding window was used. The best fitting substitution model was estimated for each gene using the Akaike Information Criterion in jModelTest 2.1.2 [53]. Single gene alignments were concatenated with GENEIOUS R6.1.8 (S1 Table). Phylogenetic analyses of single genes were conducted by Bayesian methods, while the phylogeny of the concatenated alignment was conducted using both Bayesian and maximum likelihood methods. Maximum likelihood analyses were conducted using RaxML 8.0 [54] with 5000 bootstrap replicates under the GTR + I + G model treating every gene as a separate partition. Bayesian phylogenetic analyses were conducted using MrBayes 3.2.1 [55]. Two separated analysis were performed using the default priors and the GTR + I + G model using three heated (temp = 0.2) and one cold chain per analysis. Gaps were treated as missing data and in the multi-gene analysis each gene and different Cox1 codon positions were treated as a different partitions. Analyses were run for 20 million generations, sampling trees every 500^th^ generation. Run convergence was assessed using standard deviation of split frequencies and Potential Scale Reduction Factors (PSRF). Of the results 25% were discarded as burnin and burnin size was evaluated using a generation/Log-likelihood scatterplot. Reproduction modes were traced along the Bayesian majority rule consensus tree phylogeny using maximum parsimony and maximum likelihood methods implemented in Mesquite 3.10 [56].

### Cytological staining

Egg laying females were dissected from freshly collected coffee roots, smeared on microscope slides and stained according to Triantaphyllou [57]. Smears were hydrolysed by submerging the slide for 6 minutes in 1N HCl, fixed for 30–60 minutes in freshly prepared fixative consisting of 75% absolute ethanol and 25% acetic acid, stained for 30 minutes in propionic orcein and washed using 45% propionic acid. Preparations were sealed using nail polish and oogenesis was studied using an Olympus BX51 DIC microscope (Olympus Optical). Chromosome numbers were estimated from late-prophase or early-metaphase chromosomal planes, because in these stages the chromosomes are discrete and can be counted accurately [30]. Multifocal movies were made from chromosome planes and subsequently analysed and counted frame-by-frame using ImageJ software [58]. Movies are available online at http://www.nematodes.myspecies.info and on the Dryad Digital Repository (http://dx.doi.org/10.5061/dryad.9f63r).

## Results and discussion

### Morphological identification of coffee root-knot nematodes from Tanzania

From all nine sampled locations a *Meloidogyne* species was isolated, which were morphologically similar and, based on their Cox1 sequence (see section 3.3.1), were confirmed to belong to the same species. As currently the descriptions of African coffee root-knot nematodes are limited to morphological features, the morphology of this species is compared with original type material of *M*. *africana*, *M*. *oteifae*, *M*. *megadora* and *M*. *decalineata*. For morphological comparison both a cultured population on *Coffea arabica* and field caught populations were used.

#### Females

**Fig 1:**
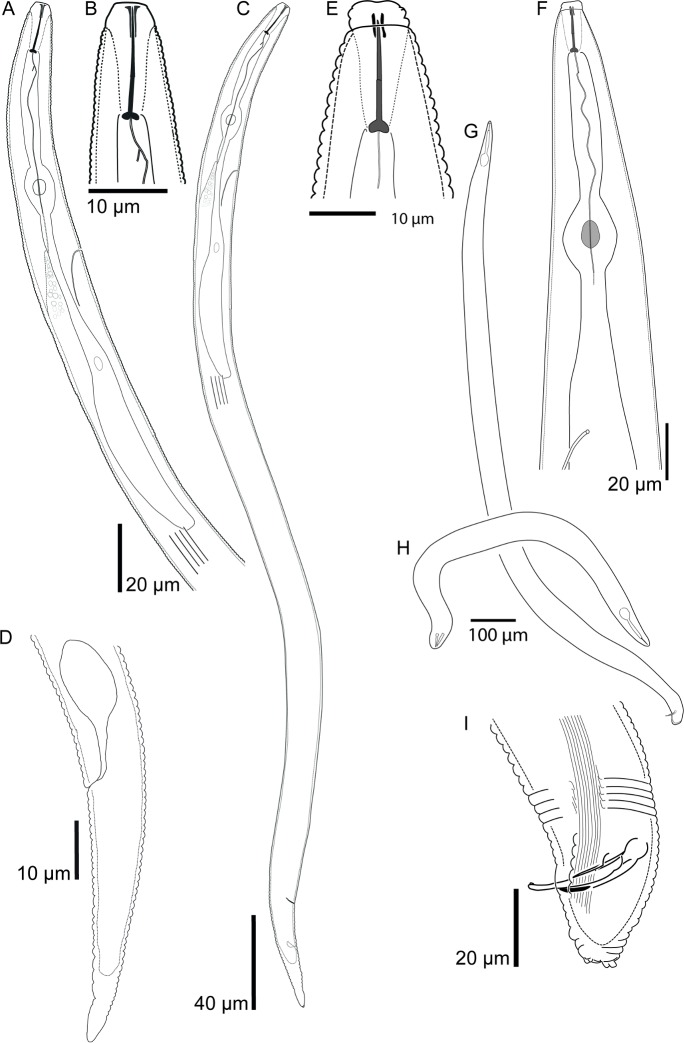
Camera lucida drawings of *Meloidogyne africana* from Tanzania. (A) second-stage juvenile anterior body; (B) second-stage juvenile head; (C) second-stage juvenile habitus; (D) second-stage juvenile tail; (E) male head; (F) male anterior body; (G, H) variable male habitus during development as sex-reversed females; (I) male tail.

**Fig 2:**
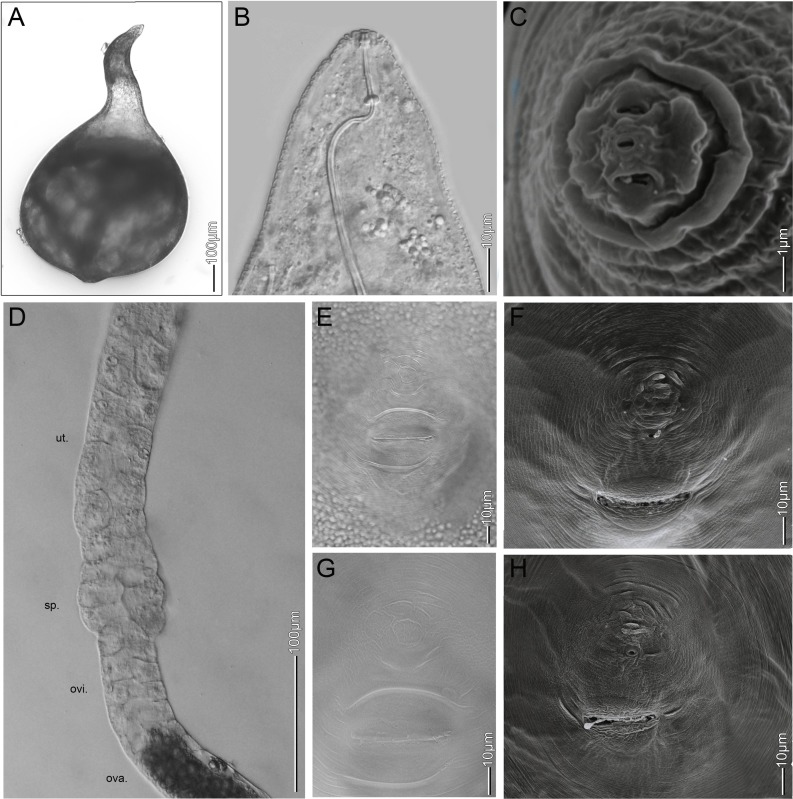
Light microscopy and SEM of *Melodigoyne africana* females. (A) general habitus with characteristic protuberance; (B) head, lateral view; (C) cephalic region (en face view); (D) gonad morphology of uterus (ut.), spermatheca (sp.), oviduct (ovi.) and ovarium (ova.); (E, G) photomicrographs of perineal pattern; (F,H) SEM photograph of perineal pattern.

A morphological and morphometric comparison of females and perineal patterns between *M*. *africana*, *M*. *oteifae*, *M*. *megadora* and *M*. *decalineata* revealed that these species are less different than previously considered (Table 1). Whitehead [18] described the female body of *M*. *decalineata* without or with a very slight protuberance, however, a clear protuberance was observed in paratype specimens. Perineal patterns of *M*. *decalineata* and *M*. *oteifae* were differentiated from *M*. *africana* by exhibiting a narrow versus a wide lateral field [17, 18], however, examination of type material revealed no significant morphological differences. *Meloidogyne oteifae* was originally further differentiated from *M*. *africana* by the circles of striae, which are crossed by other striae radiating from the vulva [17], however, the striation pattern around the vulva observed in paratype specimens of *M*. *africana* and *M*. *decalineata* was identical. Also, the morphology of the female head is very similar for all considered species (Table 1, Figs 1 and 2). Thus, no female morphological or morphometric characters separate our populations, *M*. *africana*, *M*. *oteifae*, *M*. *megadora* and *M*. *decalineata*.

**Table 1:** Comparison of female morphological and morphometric characters between *Meloidogyne* species parasitizing coffee in Africa.

	*M*. *africana* Whitehead 1959^5^	*M*. *oteifae* Elmiligy 1968	*M*. *decalineata* Whitehead 1968	*M*. *megadora* Whitehead 1968	*Meloidogyne* sp.
n	17	10	20	12	22
L	760±73 (660–910)	600 (520–680)	819±133 (649–1041)	683±87 (554–845)	615±95 (400.0–770.0)
Stylet	15	13.5 (13–14)	14 (12–17)	15 (13–17)	14.3±0.8 (13.0–15.5)
Stylet knobs width	/	/	/	3 (3–5)	3.1±0.3 (3.0–4.0)
DGO	4–9	3.5 (3–4)	4	6 (4–8)	5.7±0.8 (4.5–7.0)
a	1.6±0.16 (1.4–1.9)	/	1.6±0.20 (1.2–2.1)	1.45±0.218 (1.1–1.8)	1.6±0.2 (1.3–2.4)
Body shape	pyroid	pyroid	pyroid	pyroid	pyroid
Protuberance	present	present^1^	present^2^	mostly present	present
ES porus	16–30 annules	12–15 annules	20–50 annules	8–30 annules	14–35 annules
Phasmids	close to tail terminus	close to tail terminus	close to tail terminus	close to tail terminus	close to tail terminus
Elevated perenium	present	present^3^	present	sometimes present	present
Start lateral field	faint	faint	rudimentary lateral field in some patterns	not generally visible, posterior part wiyh short coarse striae	faint
Dorsal arch	low	low	low	low	low
Stria	very fine	very fine	very fine	very fine	very fine
Stria surrounding vulva	sometimes striae surrounding vulva	striae surrounding vulva^4^	sometimes striae between tail and vulva	/	sometimes striae surrounding vulva
Stylet knobs	rounding or tending to flatten anterior	rounded	rounded or backward sloping	back-sloped	rounded
Head annules	1 or 2 annules behind headcap	difficult to distinguish	2 annules behind headcap	3 annules behind headcap	1 or 2 annules behind headcap

^1^ Not mentioned in the original description of Elmiligy (1968) but clearly observed in paratype slides.

^2^ Whitehead (1968) mentions posterior end without or with very slight protuberance, however, we observe a clear protuberance in all paratype specimen.

^3^ Elmigly (1968) mentiones the perineal pattern not raised on a knob, however, we observe a clearly elevated perenium in several paratype perineal patterns.

^4^ Elmigly (1968) differentiated *M*. *oteifae* from *M*. *africana* by the circles of striae which are crossed by other striae radiating from the vulva and by the absence of the wide relatively clear area in the lateral field. However, we observe exactly the same striation around the vulva in several paratype specimen of *M*. *africana* and we observed no difference in morphology of the lateral field between paratype material of *M*. *oteifae* and *M*. *africana*.

^5^ Standard deviations are wrongly calculated in Whitehead (1959), standard deviations in this table are taken from Whitehead (1968).

#### Second-stage juveniles

**Fig 3:**
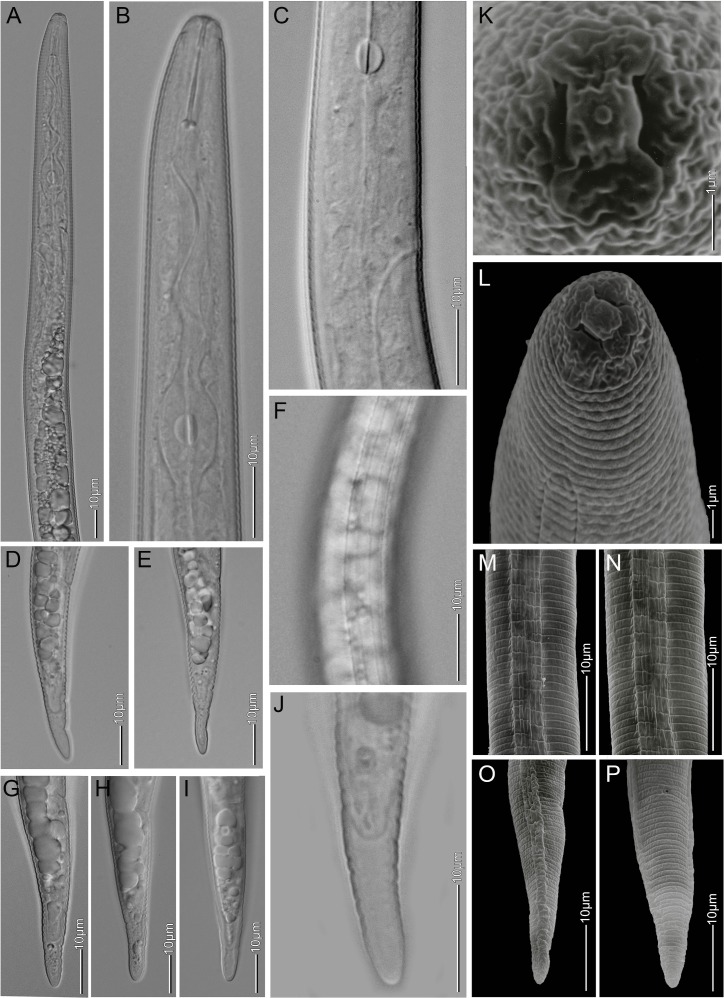
Light microscopy and SEM of *Meloidogyne africana* second-stage juveniles. (A,B) anterior body; (C) meta- and post-corpus region; (D, E, G, H, I) tail variation; (F) mid-body lateral field; (J) hyaline tail region; (K) cephalic region, en face view; (L) cephalic region, lateral view; (M, N) SEM photographs of mid-body lateral field; (O) tail, lateral view; (P) tail, ventral view.

The morphological comparison between second-stage juveniles reveals strong similarities between our population, and both M. africana and M. oteifae (Table 2). By contrast, the juvenile characteristics of M. decalineata match well with M. javanica. The total length, tail length, hyaline tail terminus of M. decalineata is markedly longer compared to M. africana, M. oteifae and our population, and the tail terminus has a subacute pointed terminus (vs rounded tail terminus). These differences are unexpected as M. decalineata was reported from the same site and host in the Lushoto district as three of our populations [16]. Furthermore, a long juvenile tail usually implies that phasmids are positioned far apart in the perineal pattern, due to swelling of the juvenile body during transition to the female live stage. In M. decalineata the phasmids are positioned close to each other in the perineal pattern (Table 1). These observations indicate that M. decalineata was most likely described from a species mixture and that the described juveniles of M. decalineata belong to M. javanica. This error is possible as M. javanica has been reported from coffee and weeds from coffee plantations in East Africa and is a commonly occurring root-knot nematode in the region [16]; personal observations TJ). Additionally, Whitehead [16] reports M. decalineata to be a very common species in the village of Lushoto, however, after profound sampling only M. africana-like juveniles were recovered during the current sampling. In conclusion, the differences between juveniles of our population (Figs 1 and 3), M. africana and M. oteifae are insignificant and the juveniles that have been described for M. decalineata [18] are here considered to belong to M. javanica. Juveniles of M. megadora have a longer tail with a shorter hyaline terminus in comparison to our population, M. africana and M. oteifae.

**Table 2:** Comparison of second-stage juvenile morphological and morphometric characters between *Meloidogyne* species parasitizing coffee in Africa.

	*M*. *africana* Whitehead 1959^7^	*M*. *oteifae* Elmiligy 1968	*M*. *decalineata* Whitehead 1968	*M*. *javanica* Eisenback and Triantaphyllou 1981		*M*. *megadora* Whitehead 1968	*Meloidogyne sp*.
n	25	30	25	90	26		30
L	420±5 (380–470)	370 (320–400)	543±24 (471–573)	488 (402–560)	451±27 (413–548)		422.0±39 (352.0–535.5)
Stylet	14.8±1.5 (12–18)	12 (11–13)	12.4±0.68 (10.7–13.7)	10–12	12.0±0.61 (10.7–13.2)		11.5±0.5 (10.5±12.5)
DGO	3	3	3.5 (3.0–4.0)^2^	3.5	3.9±1.14 (2.3–4.8)		4.0±0.6 (3.0–5.5)
Tail	40±1.7 (30–48)	around 48^3^	48±2 (44–52)	56 (51–63)	53±3 (47–58)		42.1±1.9 (39.0–46.0)
Hyaline tail terminus	7.2±1.4 (Jepson 1987)	Around 10 (Jepson 1987)	15.5±3.1 (Jepson 1987)	13.7 (9–18)	6.3±0.6 (Jepson 1987)		10.5±1.3 (8.0–13.0)
a	24.4±1.75 (22–28)	26.5 (22–29)	36.3±1.94 (32.8–40.0)	/	27.8±2.31 (23.1–32.9)		25.5±3.1 (19.5–31.1)
c	10.8±1.82 (7.3–14.3)	8 (7.5–9.2)	11.2±0.51 (10.3–12.2)	/	8.4±0.64 (7.6–11.0)		10.1±1.0 (7.8–12.7)
Tail shape	blunt rounded terminus	tail tapering to a rounded terminus	tail tapering smoothly or irregularly to subacute terminus	tail tapering smoothly or irregularly to subacute terminus^4^	tail tapering irregularly ending in a subacute variously shaped end		blunt rounded terminus
Head annules	2	difficult to distinguish	3–4	3–4	3		2
Lateral field lines	4^1^	4	4	4	4 (2 clear + 2 fainter)		4
Areolation lateral field	outer bands areolated^4^	/	outer bands areolated	outer bands areolated^4^	/		outer bands areolated
Hemizonid	/	/	above excretory pore	above excretory pore^4^	above excretory pore		above excretory pore
Phasmids	close to tail-tip	halfway or two thirds of tail	/	/	/		close to tail-tip
Stylet knobs	rounded	rounded	ovoid	ovoid^4^	rounded, back-sloped		rounded
Rectum	inflated^4^	inflated^5^	inflated^6^	inflated^4^	not inflated		inflated

^1^ Whitehead (1959, 1968) did not observed the lateral field of second-stage juveniles. We observed 4 lines in the lateral field of paratype specimen.

^2^ Whitehead (1968) did not report the DGO length of *M*. *decalineata*, from paratype material DGO length was observed to be 3.5 (3.0–4.0), n = 6.

^3^ Estimated from body length/tail length ratio.

^4^ Own observation from paratype specimen.

^5^ Elmigly (1968) reported the rectum of *M*. *oteifae* to be not inflated, however an inflated rectum was observed in paratype specimen.

^6^ Whitehead (1968) reported the rectum of *M*. *decalineata* to be not inflated, however an inflated rectum was observed in 2 paratype specimen.

^7^ Standard deviations are wrongly calculated in Whitehead (1959), standard deviations in this table are taken from Whitehead (1968).

#### Males

**Fig 4:**
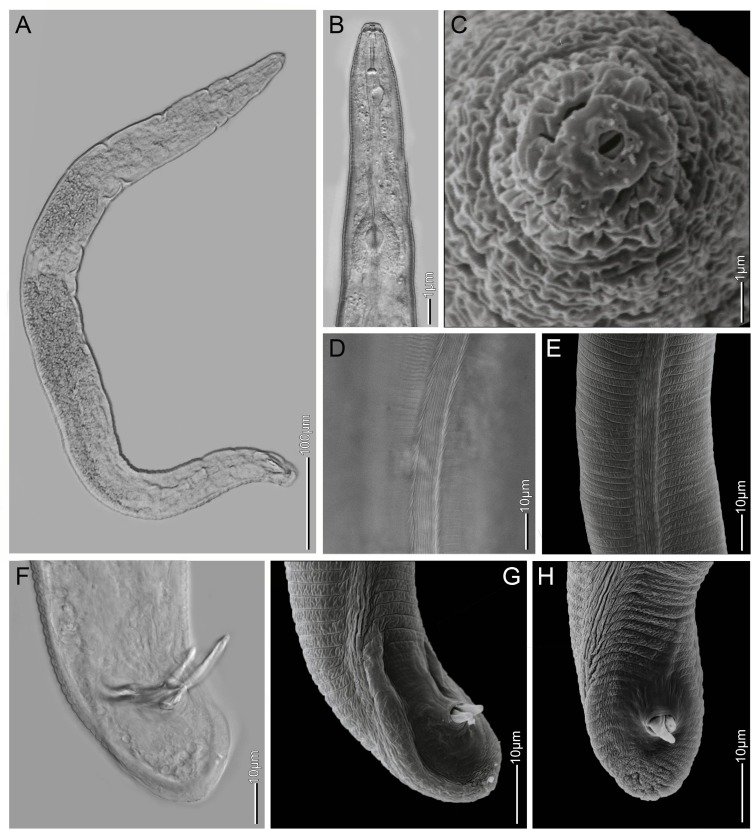
Light microscopy and SEM of *Meloidogyne africana* males. (A) habitus of dwarf sex-reversed female; (B) anterior body in lateral view; (C) cephalic region, en face view; (D, E) mid-body lateral field; (F, G, H) tail.

In both cultured and field recovered populations the males show extreme variations in shape and length, ranging from unusual partly swollen dwarf males to typical long and slender males. Their length and maximum body width varies from 816–1750 μm and 36–66 μm, respectively, resulting in a highly variable ‘a’ ratio (see Table 3, Figs 1 and 4). Several males had a second atrophied, partly developed, testis instead of a single testis. Notably, males were recovered only from dissected galls of either culture or field populations and not from soil or roots using a mistifier, Oostenbrink elutriator or modified Baermann. This would indicate that males of this species do not exit the galls and therefore are not free-living. This is further supported by empty female spermatheca, which indicates that males play no role in the reproduction of this species (see also below). The variable body shape, sexual inactivity and occurrence of intersexual features indicates a distorted development of males, which most-likely corresponds to sex-reversed females in varying stages as described for M. incognita by Papadopoulou and Triantaphyllou [59], who assumed that sex-reversal is mediated by hormonal balance and therefore greatly dependent on environmental conditions. However, we observed no differences in male development of cultured or field populations. Interestingly, similar to males from our populations, dwarf males were also reported alongside normally developed males of M. megadora by Whitehead [18], which had a reduced stylet with rounded knobs, depending on the developmental stage. This implies that the morphology of the male stylet and the body shape are of limited taxonomical use for this species since they are developmental-dependant. Surprisingly, only three male specimens are reported for M. decalineata [18], indicating that males are uncommon, possibly pointing to a similar distorted development of males.

**Table 3:** Comparison of male morphological and morphometric characters between *Meloidogyne* species parasitizing coffee in Africa.

	*M*. *africana* Whitehead 1968	*M*. *hapla* Whithead 1968	*M*. *oteifae* Elmiligy 1968	*M*. *decalineata* Whitehead 1968	*M*. *megadora* Whitehead 1968	*Meloidogyne sp*.
Male						
n	18	25	10	2	25	21
L	1470±197 (1200–1850)	1139±166 (791–1432)	1160 (980–1270)	1630, 1700	1906±330 (905–2277)	1285±245 (816.0–1750.0)
Stylet	20.7±1.08 (19–22)	20.0±1.28 (17.3–22.7)	22 (19–23)	20, 19	20.4±1.13 (18.3–21.9)	15.7±1.1 (14.0–18.0)
Stylet knobs width	/	3.5±0.52 (2.5–5.0)	/	/	5.1±0.64 (3.6–6.1)	3.5±0.4 (3.0–4.0)
DGO	(4–6)	2.9±0.23 (2.5–3.2)^1^	3.5 (3–4.5)	4	6.5±1.18 (4.0–8.3)	5±0.4 (4.0–6.0)
Spicules	(26–35)	25.7±2.42 (21.6–28.1)	33 (29–37)	33, 34, 36, 37	32.6±2.96 (25.2–36.0)	26.5±2.3 (24.0–31.0)
Gubernaculum	(7–9)	8.2±0.82 (7.2–9.4)	11 (10–12)	7	10.6±0.86 (9.4–11.9)	7.6±1.8 (6.0–10.0)
a	38.9±4.72 (31–50)	41.7±3.66 (33.3–47.0)	26 (25–28)	29.6, 42.5	52.8±7.06 (36.9–62.8)	26.0±4.0 (19.2–34.3)
Lateral field lines	4	4	4, 5 or more present in few specimen	10	mostly 4, 5 or 6 for short distance	10–13
Lateral field width	1/4 of body width	/	1/5 of body width	1/7 of body width	/	1/4 of body width
Testes	single, rarely reflexed	single, rarely reflexed	single, mostly reflexed	single	single, in 2 exceptional cases double	single, mostly reflexed

^1^ Jepson reported the DGO of *M*. *hapla* to be 4.1±1.0 (2.7–5.4).

In agreement with other similarities, all our populations exhibit a characteristic lateral field, as described for *M*. *decalineata*, i.e. narrow, occupying one-fifth of the body width, with 10 to 13 lateral lines, while the lateral fields of *M*. *oteifae* and *M*. *megadora* exhibit a variable number of lateral lines. In contrast, the lateral field of *M*. *africana* exhibits 4 lateral lines only; careful examination of the type material of *M*. *africana* furthermore reveals a remarkable similarity with males of *M*. *hapla* (Table 3). Among other characters, the DGO, stylet length, stylet knob shape, lip shape, a single short rarely reflexed testis and lateral field including typical striation observed in male type material match perfectly with *M*. *hapla*. This would indicate that the males of *M*. *africana* and *M*. *hapla* were quite conceivably mixed up by Whitehead [10], which is possible given that Whitehead [16] reported *M*. *africana* from *Pyrethrum*, a crop known to be a particularly good host of *M*. *hapla* [16, 60]. Furthermore, we also recovered *M*. *hapla* from *C*. *arabica* in Tanzania (Mufindi, Iringa) and from *Achyranthes aspera* (Mbelei, Tanga), a commonly occurring weed in coffee plantations. Indeed, *M*. *hapla* frequently occurs in the tropics at cooler higher elevations, a typical habitat for coffee cultivation in Africa, where mixed populations of *M*. *africana* and *M*. *hapla* may consequently occur. Although Whitehead [10] did not detail the extraction methods used, the fact that males of our *M*. *africana* could not be obtained using traditional mistifier, Oostenbrink elutriator or modified Baermann extraction methods supports the argument that the original males belong to a different species. For *M*. *oteifae* just one single male paratype specimen was deposited by Elmiligy [17], which does not permit for a definitive diagnosis, but which shows a remarkable similarity with *M*. *javanica* males. In conclusion, our observations indicate that males of *M*. *africana* [10] belong to *M*. *hapla*, while males from our population correspond with *M*. *decalineata* males. This further complicates the African coffee root-knot nematode taxonomic conundrum.

#### Conclusion of morphological identification

In the current study, in order to clarify the taxonomic status of African coffee root-knot nematodes, the features of females and juveniles need to be prioritised, as they are considered the superior Meloidogyne diagnostic characteristics [61]. The males of most of the investigated populations have a distorted development as sex-reversed females, and the originally described males of M. africana and M. oteifae are likely based on other species. From all of our current populations females and juveniles unequivocally match with M. africana. However, Whitehead [10] assigned males of M. hapla as the holotype of M. africana and as such and the existing type is not in taxonomic accord with M. africana. For the more recently described M. decalineata, the holotype has been correctly assigned but the juveniles have been described based on a different species. Retaining the original name M. africana and not M. decalineata will result in a greater taxonomic stability; irrespective of the current manuscript, other populations of this species will most likely be identified as M. africana, since males are rare. Hence, M. africana is not only the oldest available name but also in accord with the prevailing usage of the original name and therefore the name M. africana should be conserved. As a consequence M. decalineata is considered a junior synonym of M. africana. Reference material has been deposited in the collections of Wageningen and the Nematology Research Unit University Ghent (slides UGnem147-149).

In addition, the morphology of *M*. *oteifae* females and juveniles correspond perfectly with *M*. *africana*, while males of *M*. *oteifae* show affinity with *M*. *javanica*. Moreover, *M*. *oteifae* (accepted for publication on 24 July 1968) does not appear to have been adequately compared with *M*. *decalineata* or *M*. *megadora* published in august 1968 (accepted for publication on 10 October 1967), which were published almost simultaneously. Therefore, *M*. *oteifae* is also considered to be a junior synonym of *M*. *africana*. For *M*. *megadora*, the females and males also show remarkable similarities to our populations of *M*. *africana*, including the presence of “dwarf” males. However, *M*. *megadora* is considered to be a valid species because the juveniles have a longer tail with a shorter hyaline terminus, and the esterase isozyme profile migrates faster in comparison to *M*. *africana* [62]. Moreover, a recent molecular characterisation confirmed *M*. *megadora* to be a separate entity [63].

### Redescription of *Meloidogyne africana* (Table 4, Figs 1–4)

**Table 4:** Morphometrics of *Meloidogyne africana* cultured on *Coffea arabica*. Mean ± SD (range), all measurements in μm.

**Character**	**Females**	**Males**	**Second-stage juveniles**
N	22	21	30
L	615±95 (400–770)	1285±245 (816–1750)	422±39 (352–536)
Greatest body diam.	375±59 (300–540)	50.0±7.6 (36.0–66.0)	16.8±2.3 (14.0–22.0)
Body diam. at anus	/	/	10.2±0.7 (9.0–12.0)
Head region height	/	3.3±0.5 (2.5–4.0)	2.7±0.5 (2.0–4.0)
Head region diam.	/	8.3±0.4 (7.5–9.0)	5.5±0.3 (5.0–6.0)
Neck length	217±48.1 (120–300)	/	/
Stylet	14.3±0.8 (13.0–15.5)	15.7±1.1 (14.0–18.0)	11.5±0.5 (10.5±12.5)
Stylet knobs width	3.1±0.3 (3.0–4.0)	3.5±0.4 (3.0–4.0)	1.9±0.2 (1.5±2.0)
DGO	5.7±0.8 (4.5–7.0)	5±0.4 (4.0–6.0)	4.0±0.6 (3.0–5.5)
Ant. end to metacorpus	/	64.0±8.2 (54.0–78.0)	46.0±5.6 (38.0–61.0)
Excretory pore-ant.end	/	150±24.9 (110–197)	75.5±6.9 (55.0–84.0)
Tail	/	/	42.1±1.9 (39.0–46.0)
Hyaline tail terminus	/	/	10.5±1.3 (8.0–13.0)
Spicules	/	26.5±2.3 (24.0–31.0)	/
Gubernaculum	/	7.6±1.8 (6.0–10.0)	/
a	1.6±0.2 (1.3–2.4)	26.0±4.0 (19.2–34.3)	25.5±3.1 (19.5–31.1)
c	/	/	10.1±1.0 (7.8–12.7)
c’	/	/	4.1±0.3 (3.5–4.7)
Body L/neck L	2.9±0.4 (2.3–3.8)	/	/
(Excretory pore/L)x100	/	11.7±2.0 (8.7–15.7)	17.8±1.2 (14.0–20.4)

Subfamily Meloidogyninae Skarbilovich, 1959

Genus *Meloidogyne* Göldi, 1887

*Meloidogyne africana*, Whitehead 1960

new syn. *Meloidogyne decalineata*, Whitehead 1968

new syn. *Meloidogyne oteifae*, Elmiligy 1968

#### Material examined

*Meloidogyne africana*: holotype and 3 paratype slides from Kamaara Coffee nursery, Meru district, Kenya (Rothamsted Nematode Collection 77/17/1-77/17/4); *Meloidogyne decalineata*: holotype and 3 paratype slides from Mawingo estate, Kilimanjaro, Tanganyika (Rothamsted Nematode Collection 77/10/1-77/10/4); *Meloidogyne megadora*: holotype and 3 paratype slides from Coffee research station, Amboim, Republic of Angola (Rothamsted Nematode Collection 77/13/1-77/13/4); *Meloidogyne oteifae*: holotype and 10 paratype slides from Congo (Ghent University Museum, Zoology Collection). Three *M*. *africana* populations from Lushoto, Tanzania and six populations from Mbelei, Tanzania.

#### Females

As described for *Meloidogyne africana* by Whitehead [10]. Additionally, SEM of the perineal pattern supplements the light microscopic observations and drawings of Whitehead [10] (Figs 1 and 2) and illustrates the typical characteristics of the perineal pattern. Faint striae forming a low dorsal arch, phasmids positioned adjacent to tail terminus, start of the lateral field of variable width and composed of irregular striae, perineal pattern on a raised perineum as a consequence of a clear protuberance, and vulva surrounded by circles of striae, which are sometimes crossed by other striae radiating from the vulva.

#### Juveniles

As described for *Meloidogyne africana* by Whitehead [10]. Body length has a wider range and the stylet is slightly shorter compared to the original description (Table 4). The lateral field, which was not described in the original description, is prominent with 4 lines (Figs 1 and 3), which may be areolated in the mid-body region. SEM observations indicate that more than 4 lines can be present in the mid-body region (Fig 3), providing an indication of the origin of the multiple lined lateral field of males. Second-stage juveniles have a characteristic rounded tail terminus with a long hyaline terminus (Figs 1 and 3) [10].

#### Males

As described for *Meloidogyne decalineata* by Whitehead [18], although the morphometric values (Table 4) show a wider range compared to Whitehead [18], which were based on three specimens only (Table 3).

#### Diagnosis

*Meloidogyne africana* is characterised by a distinct elevated perineal pattern with smooth striae, phasmids positioned close together and a variable lateral field. Males are variable in size with a narrow lateral field consisting of 10–13 lateral lines. Second-stage juveniles have a rounded tail, with a characteristic hyaline region. *Meloidogyne africana* is further characterised by a unique esterase isozyme phenotype. Based on ribosomal (18S and 28S rDNA) and mitochondrial (Cox1) sequences *M*. *africana* is differentiated from all members of clade I, II and III, and *M*. *coffeicola*, *M*. *cammeliae*, *M*. *ichinohei*, *M*. *mali*, *M*. *artiellia*, *M*. *beatica* (see section below). *Meloidogyne africana* is differentiated from *M*. *megadora* by a shorter juvenile tail, a differential hyaline tail terminus, a different male lateral field and a more slowly migrating esterase isozyme profile [62]. *Meloidogyne africana* is morphologically close to *M*. *acronea* Coetzee 1956, but is differentiated by the lateral lines, stylet of the male and the different juvenile tail.

### Phylogenetic analysis, molecular diagnosis and evolutionary morphology

#### Phylogenetic analyses

**Fig 5:**
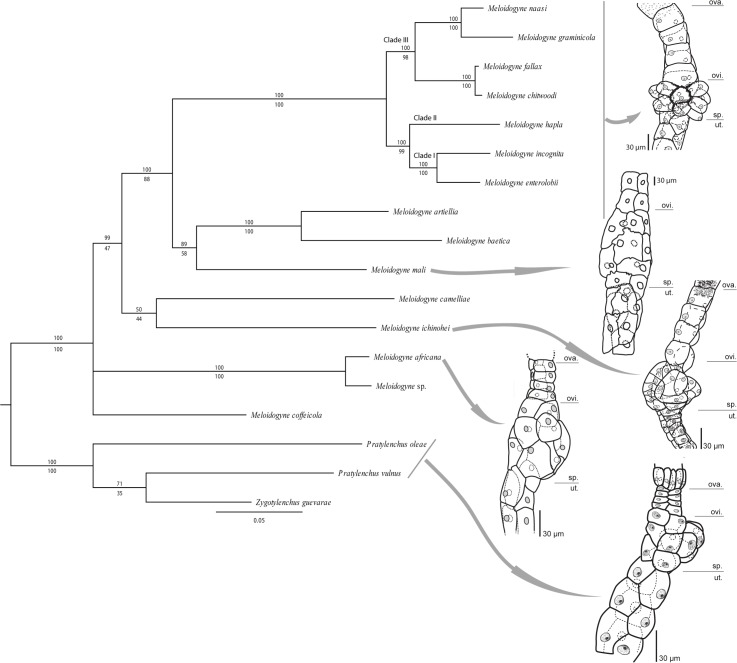
Molecular multi-gene phylogeny of the genus *Meloidogyne*. Consensus tree based on the combined nuclear 18S and 28S rDNA sequences and the mitochondrial Cox1 gene sequences. Values above branches are Bayesian posterior probabilities, values below branches are Maximum Likelihood bootstrap values. Overview of used sequences in S1 Table, for details on phylogenetic reconstruction see Materials and Methods. Evolutionary morphology of the female gonoduct is illustrated by drawings of the oviduct-spermatheca region from *Meloidogyne africana*, *Meloidogyne mali*, *Meloidogyne ichinohei*, *Meloidogyne hispanica* and *Pratylenchus thornei*; (ova.) ovaria; (ovi.) oviduct; (sp.) spermatheca; (ut.) uterus. Morphological drawing *M*. *ichinohei* and *M*. *hispanica* modified from Bert et al. (2002). *M*. *hispanica* is used as an example of typical gonoduct morphology in Clade I, II and III, only mophological exception is *M*. *microtyla* (Bert et al. 2002). Typical *Pratylenchus* gonoduct morphology is illustrated by a drawing of *Pratylenchus thornei*, modified from Bert et al. (2003).

The multi-gene sequence alignment of three genes (18S, 28S rDNA and Cox1), was 2847 base pairs in length, and is in accordance with single gene phylogenies of 18S and 28S rDNA [24, 27, 64]. The phylogenetic analysis revealed M. africana, Meloidogyne sp. and M. coffeicola to be outside of the three major clades (clade I, II and III) (Fig 5) based on the Bayesian analysis (posterior probability = 99) [24]. According to the maximum likelihood analysis, M. ichinohei, M. camellieae are also positioned outside these three major clades. Overall relationships between M. africana, M. coffeicola, M. ichinohei and M. camelliae are poorly supported and will need a phylogenomic approach in order to be resolved. The multi-gene phylogenetic analysis confirmed M. africana to be the sister taxon of an undescribed Meloidogyne species from Sansevieria sp. [27], differing in 11 base pairs (0.7%) in 18S and 35 base pairs (7.5%) in the Cox1 fragment. In comparison to other root-knot nematodes the observed 7.5% Cox1 and 0.7% 18S rDNA divergence clearly constitute a different species [46, 65], especially as 18S rDNA usually does not show sequence divergence for closely related species [65, 66].

**Fig 6:**
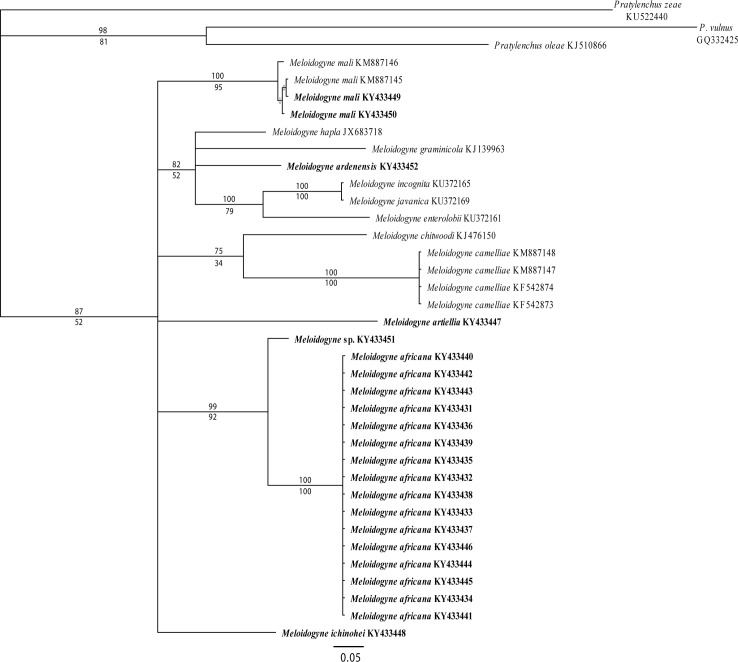
Majority rule consensus tree based on mitochondrial Cox1 sequences. Values above branches are Bayesian posterior probabilities, values below branches are Maximum Likelihood bootstrap values. For details on phylogenetic reconstruction see Materials and Methods. Genbank accession numbers are displayed behind the species name. Newly generated sequences are highlighted in bold.

The Cox1 multiple sequence alignment included 37 Cox1 sequences and was 432 base pairs in length. This alignment revealed only a single haplotype of *M*. *africana* based on the 16 sequenced specimens (8 females, 4 males and 4 juveniles) (Fig 6) and confirms that males, females and juveniles of different populations belong to the same species, *M*. *africana*. From five localities a single female was sequenced, from three locations a juvenile, a male and a juvenile were sequenced and from one location a male and a juvenile were sequenced. The presence of only one *M*. *africana* Cox1 haplotype in nine farms from Mbelei and Lusotho (Tanzania) indicates that intraspecific variability is low and that this species can be reliably identified using Cox1 DNA barcoding. However, a more extensive geographic sampling should be conducted in order to assess interspecific variability of *M*. *africana*, although recent studies show that most *Meloidogyne* spp. display little within-species genetic variability in Cox1 [46, 65].

**Fig 7:**
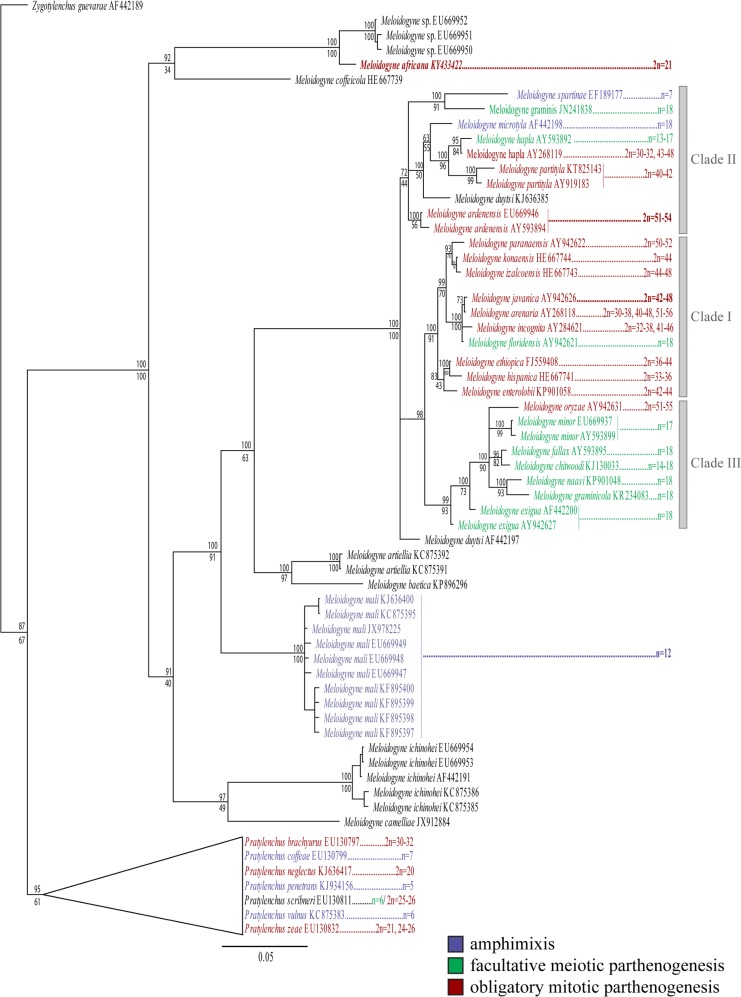
Majority rule consensus tree based on 18S ribosomal rDNA sequences with karyology. Values above branches are Bayesian posterior probabilities. Known karyotypes are displayed, reviewed by Chitwood and Perry (2009), newly generated karyotypes are highlighted in bold. Note there is no direct link between nucleotide sequences from NCBI and karyotyped populations. Amphimictic species highlighted in purple, facultative meiotic parthenogenetic species highlighted in green, mitotic parthenogenetic species highlighted in red.

In order to differentiate *M*. *africana* from a broader range of *Meloidogyne* species a 18S rDNA single gene phylogeny was constructed (Fig 7), based on a multiple sequence alignment of 1819 base pairs in length. The phylogenetic tree is in accordance with previous 18s rDNA based phylogenies [24, 27, 64] and confirms the species identity of *M*. *africana* and its position outside the 3 major clades of the genus.

Also, three ITS1-5.8S-ITS2 rDNA sequences were deposited on GenBank (accession numbers: KY433428-KY433430) because this remains a frequently used region for identification of plant-parasitic nematodes [63, 67]. However, ITS has been described to exhibit multiple highly divergent copies in a single *Meloidogyne* individual due to the suggested hybrid origin of these species [63, 68]. Since *M*. *africana* is also suggested to be a triploid species (see section below), ITS is considered to be unreliable for identification purposes. Furthermore, ITS sequences are extremely variable among root-knot nematodes [68, 69] and multiple sequence alignments are unlikely to generate homologous nucleotide positions needed for a reliable phylogenetic analyses.

#### Evolutionary morphology of the perineal pattern and protuberance

The perineal region of Meloidogyne africana appeared to show an ancestral pattern because it resembles a pre-adult of M. javanica [10] and this proposed ontogenetic pattern appears to correspond with the obtained phylogeny. In this regard, Karssen [70] already noted that perineal patterns of all Meloidogyne pre-adults significantly differ from those of mature females because the pre-adult remains enclosed in the juvenile cuticle. Given that perineal patterns of pre-adult M. javanica (and other Meloidogyne spp.) are hemispherical-shaped, with a fine striation, they resemble the perineal pattern of M. africana adults. However, the hemispherical shape of the perineal pattern is directly linked to the presence of a protuberance in Meloidogyne species, which is mostly associated with a finely striated perineal pattern (exceptions are M. graminis (Sledge & Golden, 1964) Whitehead, 1968, M. marylandi Jepson & Golden in Jepson, 1987 and M. sasseri Handoo, Heuttel & Golden, 1994, which show a protuberance in combination with a coarsely striated perineal pattern) [70]. This finely striated, hemispherical perineal pattern is most likely the result of less body expansion during the transition towards adult, which has evolved several times independently from each other, as has the presence of a protuberance. Thus, it is unclear if the finely striated perineal pattern and a protuberance of M. africana, M. ichinohei and M. coffeicola represents the ancestral state of the genus given that M. camelliae, M. mali, M. beatica Castillo, Vovlas, Subbotin & Trocolli, 2003 and M. artiellia show a coarsely striated perineal pattern without protuberance. Yet, incompletely swollen species with a protuberance might be considered as an intermediate step between vermiform nematodes of the genus Pratylenchus and completely globular nematodes of the genus Meloidogyne.

#### Evolutionary morphology of the female gonoduct

As in all Tylenchina, the two gonoduct branches of M. africana and M. mali each consist of a uterus, a spermatheca, an oviduct and an ovary (Figs 2 and 5). The uterus of M. africana and M. mali is a very long tricolumella with multiple cells as in all other Meloidogyne spp. [43] but distinct from the short uterus of most Pratylenchidae [71]. This extended uterus convergently evolved in endoparasitic nematodes, including entomopathogenic steinernematids, false root-knot nematodes (Naccobus spp.), cyst- and root-knot nematodes [45]. The spermatheca of M. africana is not offset, consisting of 12–14 cells, without lobe-like cells and without interlaced cell boundaries (Figs 2 and 5). Also the spermatheca of M. mali is not offset, without lobe-like cells, consisting of 13–16 cells (Fig 5), and cell boundaries are slightly interlaced but less interlaced than in M. microtyla [43]. Among root-knot nematodes the spermatheca morphology of both M. africana and M. mali is similar to M. microtyla and M. ichinohei in lacking lobe-like cells and clearly inter-laced cell boundaries, while in most other Meloidogyne spp. the spermatheca is composed of 16–18 lobe-like cells with interlaced boundaries [43]. Based on the obtained phylogeny, a spermatheca comprised of a limited number of cells, with not-interlaced and not-lobe like cells, could be an ancestral feature within the genus Meloidogyne because it is remarkably similar to spermatheca morphology of Pratylenchus [71]. In both M. africana and M. mali the oviduct consists of two rows of four cells, characteristic for all Pratylenchidae and most Tylenchomorpha [43, 45, 71, 72].

### Isozyme electrophoresis

**Fig 8:**
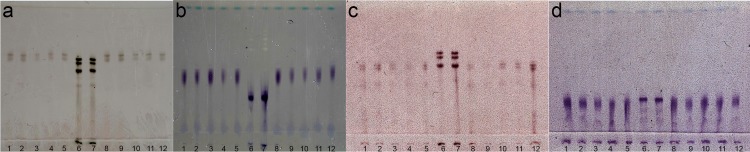
Isozym profiles of *Meloidogyne africana* and *Meloidogyne ichinohei*. Lane 6 and 7 represent *Meloidogyne javanica* reference phenotypes, lane 1–5 and 8–12 represent phenotypes of *Meloidogyne africana* and *Meloidogyne ichinohei*. (a) esterase AF2 phenotype of *Meloidogyne africana*; (b) malate dehydrogenase H1 phenotype of *Meloidogyne africana*; (c) esterase IC2 phenotype of *Meloidogyne ichinohei*; (d) malate dehydrogenase N1 phenotype of *Meloidogyne ichinohei*.

Electrophoretic isozyme analysis of single young egg-laying females of *M*. *africana* revealed a unique esterase phenotype, consisting of two fast migrating esterase bands, designated as AF2 (Fig 8). The first is in approximately the same position as the fastest migrating *M*. *javanica* reference band (61.7%) and the second at 65.7% migrating speed. This phenotype resembles the slightly slower migrating M2-VF1 phenotype of *M*. *artiellia* [70] and the faster migrating C2 and Me3 phenotypes of respectively *M*. *coffeicola* [73] and *M*. *megadora* [62]. The malate dehydrogenase isozyme analysis revealed a single broad band (migrating speed 45.3%) in a similar position as the H1 phenotype of *M*. *hapla*.

In order to obtain isozyme information for more *Meloidogyne* species, the isozyme phenotypes of *Meloidogyne ichinohei* were studied, revealing another unique esterase phenotype. This phenotype consists of two clear slow migrating bands (relative migrating speed 53.0 and 56.1%), herein designated IC2 (Fig 8). The malate dehydrogenase isozyme analysis revealed a single broad band corresponding to the commonly occurring N1 phenotype. It has been observed that both the malate dehydrogenase isozyme analysis of *M*. *africana* and *M*. *ichinohei* are slightly smeared and have a broader appearance than the H1 and N1 phenotype respectively. This smeared appearance was not caused by an analysis artefact as it was verified in several separate analyses using different populations. Interestingly, both the *M*. *coffeicola* C1 phenotype [73] and the *M*. *artiellia* N1b [70] phenotype also have a slightly smeared appearance implying that these differences could be informative and characteristic for *Meloidogyne* spp. outside the three major clades. These results indicate that *M*. *africana* and *M*. *ichinohei* can be reliably identified using isozyme electrophoresis, confirming that isozyme electrophoresis remains a highly reliable, if not labor intensive, diagnostic tool for root-knot nematodes [46, 69, 74].

### Evolution of reproduction and oogenesis

#### Meloidogyne javanica

To re-establish the propionic orcein staining protocol of Triantaphyllou (1985), M. javanica reference material originating from Spain, F1836-3 [46], collected and maintained on S. lycopersicum was stained and compared to the M. javanica observations of Triantaphyllou [75]. Based on 8 late prophase to early methaphase chromosomal planes, our M. javanica population appeared to have 2n = 44 chromosomes, identical to two M. javanica populations karyotyped by Triantaphyllou [75] originating from England and Australia. Oocytes approaching the consistently empty spermatheca were observed to exhibit 44 univalent chromosomes, indicating that chromosome pairing did not take place during the zygotene stage, and therefore indicating that reproduction occurred by mitotic parthenogenesis.

#### Meloidogyne africana

Several mitotic divisions of the oogonia took place in the apical germinal zone. From 20 favourable late-prophase or early-metaphase chromosomal planes of these mitotic divisions, the chromosome number of M. africana was determined to be 2n = 21. Similar to other Meloidogyne spp., the chromatin of young oocytes in the synapsis zone was found to be strongly orcein-stained [30]. In the maturation zone, oocytes progressively were seen to increase in size, while chromatin in this region were only weakly stained. When matured oocytes approached the spermatheca, 21 univalent chromosomes were observed in four chromosomal planes at prophase, indicating that chromosome pairing did not take place during the zygotene stage. This observation, together with the spermatheca being consistently empty and the fact that the males were sexually inactive (see above), indicates that reproduction takes place by mitotic parthenogenesis. This is the first report of a Meloidogyne species with a chromosome complement of 21. All other obligatory mitotic parthenogenetic Meloidogyne spp. are known to have at least 2n = 30 chromosomes [76]. Thus, M. africana constitutes the root-knot nematode with the lowest known number of chromosomes to reproduce by mitotic parthenogenesis. Interestingly, this mitotic parthenogenetic mode of reproduction correlates with the distorted development of sex-reversed females and the sexual inactivity of M. africana males, since this behaviour has so far been found only in mitotic parthenogenetic species [59]. However, these dysfunctional males are nevertheless thought to play an important role in ecological adaptation, as they reduce the population density in successive generations of parthenogenetic root-knot nematodes [29, 77].

#### Meloidogyne ardenensis

A M. ardenensis population was collected from the roots of Ligustrum sp. (Wageningen, The Netherlands; GPS coordinates: 51.975688, 5.675987) in early spring, 2016, when young egg-laying females were present. The population was identified using both the morphology of juveniles and 28S rDNA and mitochondrial COX1 sequences. Similar to other Meloidogyne spp., several mitotic divisions were observed in the germinal zone. From 10 favourable late-prophase or early-metaphase planes of these mitotic divisions, the chromosome number of M. ardenensis was determined to be 2n = 51–54, with the variation in chromosome number most likely due to the difficulty in the counting process, since chromosomes of M. ardenensis are small and often positioned extremely close to one another. The mature oocytes approaching the spermatheca were found to comprise approximately 54 univalent chromosomes, with no oocytes observed with a haploid chromosome number, indicating only mitotic divisions and reproduction by mitotic parthenogenesis. This is in agreement with the high chromosome number of M. ardenensis, given that all polyploid species with more than 40 chromosomes reproduce by mitotic parthenogenesis [30, 37, 75, 76, 78, 79]. Despite being parthenogenetic, males of M. ardenensis appear to be sexually active, as spermatheca in all specimens studied were filled. However, it is not uncommon that the spermatheca of mitotic parthenogenetic species are filled with sperm within the genus Meloidogyne [30, 37, 75]. In the case of M. javanica and M. hapla race B, a spermatozoon is even able to enter the oocyte, but the spermatozoon degenerates within the oocyte and fertilization does not occur [37, 75].

#### Meloidogyne mali

A *M. mali population was collected from the roots of Ulmus* sp. originating from the field trial “Mierenbos” (Wageningen, The Netherlands; GPS coordinates: 51.979623, 5.706362), a location previously sampled by Ahmed et al. [64]. The population was identified as *M. mali* based on juvenile and female morphology and on Cox1 DNA sequences. Young egg-laying females were collected from the field and directly used for cytogenetic staining. From 2 favourable late-prophase chromosome planes studied during mitotic divisions in the apical part of the germinal zone, the diploid chromosome number of *M. mali* was determined as 2n = 24. After the oocytes increased in size in the maturation zone, 6 oocytes were observed with 12 bivalent chromosomes, showing that meiosis was taking place. In all of the specimens studied, the spermatheca was clearly filled with sperm, and in several eggs the inclusion of a sperm nucleus was observed. In one egg, the fusion between the small sperm nucleus and the larger egg nucleus was observed. These observations reveal that *M. mali* reproduces by amphimixis in the presence of males. However, no females with empty spermatheca have been isolated, and it remains to be confirmed whether *M. mali* is also capable of meiotic parthenogenesis. *Meloidogyne mali* is the first *Meloidogyne* spp. to be identified with a haploid chromosome complement of n = 12, demonstrating that chromosome numbers are even more variable than previously anticipated [76].

#### Evolution of reproduction and oogenesis in root-knot nematodes

**Fig 9:**
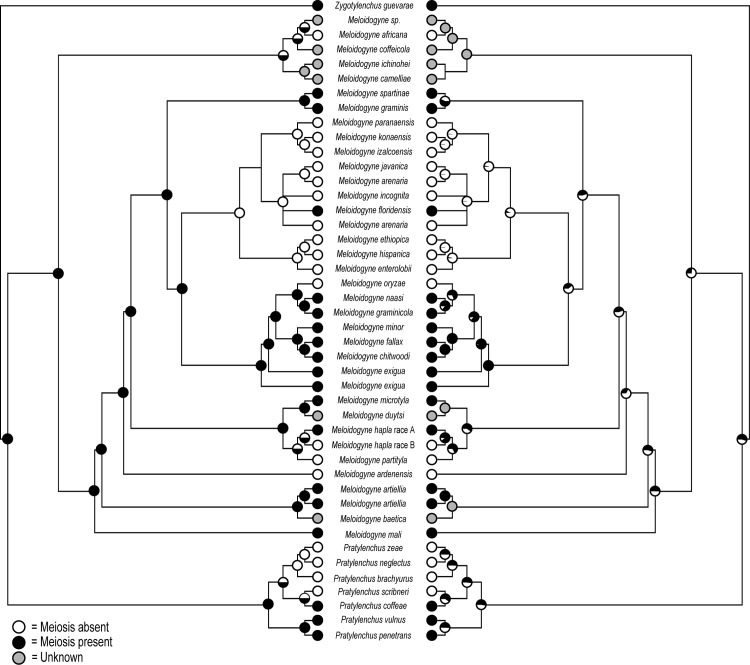
Maximum parsimony (left) and maximum likelihood (right) ancestral state reconstructions of the evolution of mitotic parthenogenesis and the associated loss of meiosis. Pie charts on internal nodes indicate the likelihoods of the different character states at each node and grey nodes indicate unknown character states. Presence and absence of meiosis are visualised in black and white, respectively.

In order to elucidate the evolution of reproduction and oogenesis within the genus Meloidogyne, chromosome number and reproduction mode of Meloidogyne spp. were plotted on the 18S rDNA phylogenetic tree (Fig 7). The results provide a completely new understanding on the evolution of reproduction within the genus Meloidogyne. To begin with, mitotic parthenogenesis has evolved independently on five occasions according to the maximum parsimony ancestral state reconstruction (Fig 9), being present in all three major clades and in M. africana. This evolutionary pattern is less resolved in the maximum likelihood ancestral state reconstruction as most of the ancestral nodes remain unresolved. In either case, our results contradict the traditional hypothesis that mitotic parthenogenetic species are restricted to one clade [29, 80]. The presence of mitotic parthenogenesis in Meloidogyne africana was unexpected, as Meloidogyne spp. in this part of the tree were previously understood to reproduce by meiotic parthenogenesis [25]. The presence of mitotic parthenogenesis within clade II, as observed for M. ardenensis, is in line with the observation of mitotic parthenogenesis in M. hapla race B [30, 37, 81] and M. partityla (2n = 40–42) [36, 82]. Curiously, the mitotic parthenogenetic species M. oryzae (2n = 51–55) and M. ardenensis (2n = 51–54) both have a triploid genomic composition, in contrast to their closest phylogenetic relatives, which are facultative meiotic parthenogenetic, and have three times less chromosomes, namely n = 18 (Fig 7). Similarly, in Clade I M. inornata Lordello, 1956 (2n = 54–58) and some populations of M. arenaria (2n = 51–56) appear to have a triploid genomic composition, in comparison with M. floridensis (n = 18), the only meiotic parthenogenetic species within Clade I. However, most species in Clade I do not have an exact chromosome complement of 18, probably as a consequence of aneuploidy and polysomy, as well as structural chromosome rearrangements [25]. These drastic changes in chromosome number are to be expected since root-knot nematodes, and indeed most other nematodes, have holocentric chromosomes with a diffuse centromere activity [83, 84]. Furthermore, it is certain that not all mitotic parthenogenetic Meloidogyne spp. have triploid genomes, with some populations of M. microcephala and M. hapla reported to be tetraploid [79, 85].

These observations suggest that a triploid genomic composition is associated with mitotic parthenogenetic reproduction, as has already been observed in several Heteroderidae. In their case, the basic chromosome number of amphimictic species is assumed to be n = 9, with *Heterodera trifolii* Goffart, 1932 (3n = 24–28), *H*. *lespedezae* Golden & Cobb, 1963 (3n = 27) and *H*. *sacchari* Luc & Merny, 1963 (3n = 27) representing triploid mitotic parthenogenetic species [86]. This has also been reported for a wide range of animals and plants [87]. If mitotic parthenogenetic species have indeed evolved several times independently triggered by a triploid genome, it is also likely that *M*. *africana* has a triploid genomic composition. This would imply that *M*. *africana* evolved from an ancestral species with n = 7 chromosomes. Interestingly, *M*. *spartinae* (Clade II) and *M*. *kikuyensis* have been both characterized as having n = 7 chromosomes [31, 32]. It should also be noted that the chromosome number of *M*. *kikuyensis* and *M*. *spartinae* was mistakenly reported as n = 9 in Castagnone-Sereno et al. [25]. Interestingly, *M*. *kikuyensis* is probably also positioned outside the three major clades as the spermatheca has a less pronounced lobe-like shape [31] and because of the deviating male head and juvenile tail morphology [15, 88, 89]. This phylogenetic placement was confirmed by preliminary molecular data (Eisenback J.D. personal communication).

The phylogenetic positions of the mitotic parthenogenetic *M*. *africana* with 2n = 21 chromosomes (Fig 7) and the amphimictic *M*. *kikuyensis* with n = 7 chromosomes favour the hypothesis of Triantaphyllou [30, 32] that sexual *Meloidogyne* spp. could lie close to the ancestry of the genus, and that mitotic parthenogenetic species evolved from them. Indeed the maximum parsimony ancestral state reconstruction shows meiosis to be present on this node, while the maximum likelihood ancestral state reconstruction is resolved equivocal. Yet, this hypothesis is consistent with the restricted chromosome numbers of the amphimictic *Pratylenchus penetrans* Cobb, 1917 (n = 5), *P*. *vulnus* Allen & Jensen, 1951 (n = 6) and *P*. *coffeae* (Zimmerman, 1898) Filipjev & Schuurmans Stekhoven, 1942 (n = 7) [90]. Furthermore, in the most closely-related genus *Pratylenchus*, mitotic parthenogenetic reproduction also appears to have evolved independently on several occasions, based on the chromosome numbers of *P*. *zeae* Graham, 1951 (2n = 24–26), *P*. *scribner*i Steiner, 1943 (2n = 25–26), *P*. *neglectus* (Rensch, 1924) Filipjev and Schuurmans Stekhoven, 1941 (2n = 20) and *P*. *brachyurus* (Godfrey, 1929) Filipjev & Schuurmans Stekhoven, 1941 (2n = 32) [90] that are in an unrelated phylogenetic position [91]. This indicates that the basic haploid chromosome number of the genus *Meloidogyne* could possibly be n = 7, as in *P*. *coffeae*, from which mitotic parthenogenetic species could have evolved with a triploid genome (3n = 21). These triploid genomes are most likely the product of reticulate evolution through genome duplication, introgression or hybridization events.

However, based on this hypothesis it remains unclear how chromosome numbers evolved from n = 7 to n = 18 within the genus *Meloidogyne* [30]. We propose two hypotheses: either this higher chromosome number was established by polyploidy, or alternatively it was established through fragmentation of chromosomes. While these hypotheses are not mutually exclusive, the latter idea appears the more likely, as chromosomes of *M*. *kikuyensis* are significantly larger than chromosomes of other *Meloidogyne* spp. [31]. This is a particularly attractive possibility, given that *M*. *kikuyensis* was occasionally observed to have an extra small chromosome that could itself have fragmented from another chromosome [31]. Moreover, the absence of centromere activity in *Meloidogyne* spp. indicates that fragmentation of chromosomes may occur more often than in other organisms with centralized centromere activity [83, 84]. Fragmentation also appears to have an evolutionary advantage over losing chromosomes, since it ensures that genetic material continues to be passed on to the next generation. Also, the fact that some *Meloidogyne* spp. have a large variation in chromosome number between n = 7 and n = 18 (*M*. *hapla* race A n = 13–17, *M*. *chitwoodi* n = 14–18 and *M*. *minor* n = 17), as well as the phylogenetic position of *M*. *mali* (n = 12) with an intermediate chromosome complement between n = 7 and n = 18 serve to add support to this hypothesis [76]. Chromosome fragmentation or aneuploidy is already known for the nematode genus *Cactodera* (Heteroderidae), with a basic chromosome number of amphimictic species of n = 9 having evolved to n = 12–13 in the meiotic parthenogenetic *Cactodera betulae* through chromosome fragmentation or aneuploidy [86]. Similar processes have been documented in several insect species, such as Lepidoptera and Hemiptera [92, 93].

### Bionomics

**Fig 10:**
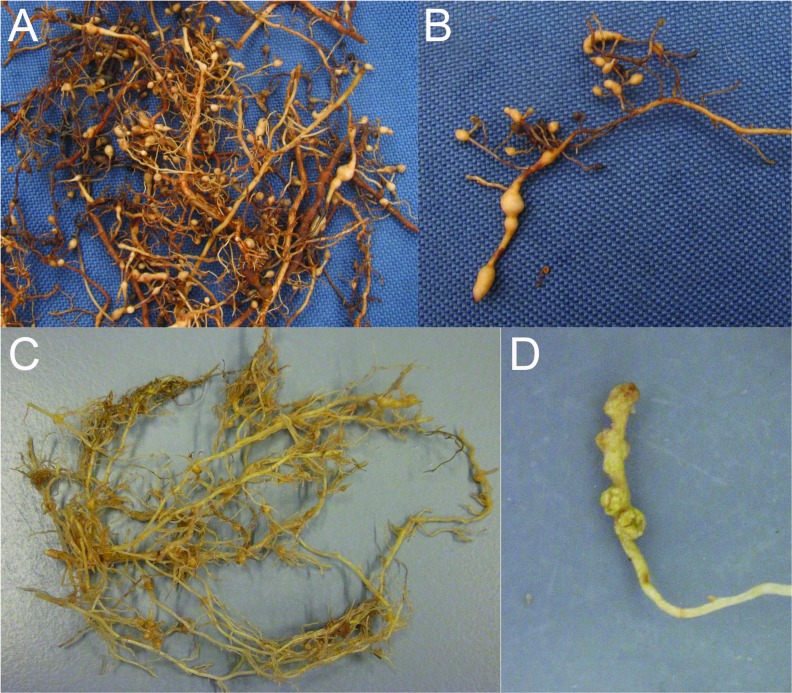
Symptoms caused by *Meloidogyne africana* on *Coffea arabica* and *Solanum lycopersicum* (cv. Moneymaker). (A) overview of galling on *Coffea arabica*; (B) detail of typical root-tip galls on *Coffea arabica*; (C) overview of galling on *Solanum lycopersicum;* (D) detail of galls on *Solanum lycopersicum*.

Host tests revealed that *M*. *africana* was not only able to parasitize *C*. *arabica* but also *S*. *lycopersicum* (cv. Moneymaker) and *Sansevieria* sp. The above-ground symptoms include stunting, chlorosis and necrosis of leaves. In coffee roots, galls are usually positioned on the apical tip of the root, resulting in the impediment of root extension (Fig 10). Young galls are rounded, while older galls tend to be oval, 1–3 mm in size, and contain more than one female with egg sacs always embedded within the gall. This internal egg sac resembles *M*. *ichinohei* [94] Older roots often display cracking, sometimes causing the eggs to be expelled from the galls, an infection symptom also described for *M*. *coffeicola* [95], another species known to infect *Coffea* spp. in South America. On tomato roots, the symptoms caused by *M*. *africana* vary slightly: galls are much smaller and are more evenly distributed throughout the root system, with root tip galls less frequent. In general, the infection was less aggressive compared to coffee plants, indicating that tomato is less suitable as a host of *M*. *africana*. Interestingly, the galls of *M*. *exigua* have also been described as occupying a terminal position on coffee roots [96], suggesting that this symptom might be host-dependent. Appearance and position of galls has been used as a taxonomic feature in many *Meloidogyne* spp. descriptions [22], although our results for *M*. *africana* indicate that many of these features (size, position) are host-dependent. Interestingly, no functional males were observed for *M*. *africana* on all three hostplants (*C*. *arabica*, *S*. *lycopersicum* and *Sansevieria* sp.). Other reported hosts of *M*. *africana* include *Z*. *mays* in India [14], *C*. *annuum* in Sudan [13] and *Chrysanthemum cinerariaefolium* L., *Z*. *mays*, *Vigna Catjang* (L.) Walp., *Syzygium aromaticum* (L.) Merrill & Perry, *C*. *arabica* and *Solanum tuberosum* L. in East Africa [16]. Although these reports, based on morphological diagnostics, need to be confirmed, together with the results of our host-range test, they show that *M*. *africana* is a polyphagous species. Since *M*. *mali* and *M*. *camelliae*, are also confirmed polyphagous species [28, 64, 97], this contradicts the hypothesis that *Meloidogyne* spp. show a tendency towards polyphagy, from the basal towards the more distally-positioned [27]. This hypothesis, however, stems from limited phylogenetic-diverse sampling of root-knot nematodes, together with limited host range tests. Recent sampling and sequencing efforts [26, 28] have revealed additional species outside the three major clades, signifying that a much larger phylogenetic biodiversity in root-knot nematodes can be expected, which is the key to fully understanding the evolution of the genus.

## Supporting information

S1 Table Genbank accession numbers from sequences used to construct the concatenated phylogenetic analysis. Newly generated sequences in bold.
(DOCX)
