# Supplementary material for: Integrative taxonomy of root-knot nematodes reveals multiple independent origins of mitotic parthenogenesis
Source: PLoS One. 2017 Mar 3;12(3):e0172190. doi: 10.1371/journal.pone.0172190 (PMC5336219; doi:10.1371/journal.pone.0172190)
Supplement: S1 Table — Newly generated sequences in bold. (DOCX) [file pone.0172190.s001.docx]

S1 table: Genbank accession numbers from sequences used to construct the concatenated phylogenetic analysis. Newly generated sequences in bold.

| Species name | 18S | 28S | Cox1 |
| --- | --- | --- | --- |
| *Zygotylenchus guevarae* | AF442189 | JX261956 | / |
| *Pratylenchus oleae* | KJ510864 | KJ510856 | KJ510866 |
| *P. vulnus* | KC875383 | KP161616 | GQ332425 |
| *Meloidogyne ichinohei* | AF442191 | **KY433427** | **KY433448** |
| *M. africana* | **KY433422** | **KY433423** | **KY433434** |
| *M. artiellia* | KC875392 | **KY433426** | **KY433447** |
| *M. mali* | KJ636400 | JX978227 | **KY433449** |
| *M.* sp. | EU669950 | / | **KY433451** |
| *M. baetica* | KP896296 | AY150369 | / |
| *M. camelliae* | JX912884 | KF542870 | KM887148 |
| *M. coffeicola* | HE667739 | / | / |
| *M. hapla* | AY593892 | KF430798 | JX683718 |
| *M. incognita* | AY284621 | KP901085 | KU372164 |
| *M. fallax* | AY593895 | KC241974 | / |
| *M. graminicola* | KR234083 | KR234084 | KJ139963 |
| *M. chitwoodi* | KJ130033 | KC287193 | KJ476150 |
| *M. enterolobii* | KP901058 | KP901079 | JX683716 |
| *M. naasi* | KP901048 | KP901069 | KM491211 |
